# Supplementary material for: Synthesis, Characterization, and Anticancer Activity of Phosphanegold(i) Complexes of 3-Thiosemicarbano-butan-2-one Oxime
Source: Biomedicines. 2023 Sep 12;11(9):2512. doi: 10.3390/biomedicines11092512 (PMC10525815; doi:10.3390/biomedicines11092512)
Supplement: Supplementary file 1 [file biomedicines-11-02512-s001.zip › biomedicines-2545771-supplementary.pdf]

Article

Synthesis, Characterization, and Anticancer Activity of Phosphanegold(i) Complexes of 3-Thiosemicarbano-butan-2-one Oxime

Sani A. Zarewa [1,†], Lama Binobaid [2,†], Adam A. A. Sulaiman [1,3,*], Homood M. As Sobeai [2], Moureq Alotaibi [2], Ali Alhoshani [2] and Anvarhusein A. Isab [1,4,*]

[1] Department of Chemistry, King Fahd University of Petroleum and Minerals, Dhahran 31261, Saudi Arabia; g202101570@kfupm.edu.sa

[2] Department of Pharmacology and Toxicology, College of Pharmacy, King Saud University, Riyadh 11451, Saudi Arabia; 442202864@student.ksu.edu.sa (L.B.); hassobeai@ksu.edu.sa (H.M.A.S.); mralotaibi@ksu.edu.sa (M.A.); ahoshani@ksu.edu.sa (A.A.)

[3] Core Research Facilities (CRF), King Fahd University of Petroleum and Minerals, Dhahran 31261, Saudi Arabia

[4] Interdisciplinary Research Center for Advanced Materials, King Fahd University of Petroleum and Minerals, Dhahran 31261, Saudi Arabia

* Correspondence: adamahmed@kfupm.edu.sa (A.A.A.S.); aisab@kfupm.edu.sa (A.A.I.)

† These authors contributed equally to this work.

Citation: Zarewa, S.A.; Binobaid, L.; Sulaiman, A.A.A.; Sobeai, H.M.A.; Alotaibi, M.; Alhoshani, A.; Isab, A.A. Synthesis, Characterization, and Anticancer Activity of Phosphanegold(i) Complexes of 3-thiosemicarbano-butan-2-one Oxime. *Biomedicines* **2023**, *11*, x. https://doi.org/10.3390/xxxxx

Academic Editor: Zhi Shi

Received: 22 July 2023
Revised: 23 August 2023
Accepted: 4 September 2023
Published: 12 September 2023

Copyright: © 2023 by the authors. Submitted for possible open access publication under the terms and conditions of the Creative Commons Attribution (CC BY) license (https://creativecommons.org/licenses/by/4.0/).

TABLE OF CONTENTS

Supporting Table .... **Table S1-S3**

FT-IR spectra data .... **Figure S1-S5**

NMR spectra data .... **Figure S6-S19**

Supplementary figures .... **Figure S20-S21**

Table S1: CHNS analysis of ligand and gold(I) complexes **1-4**

Complex	Found (Calculated) %			
	%C	% H	%N	%S
$C_5H_{10}N_4SO$ (Ligand) (**TBO**)	34.75(34.47)	5.39(5.79)	31.50(32.16)	18.22(18.40)
$[(CH_3)_3PAu(I)(C_5H_{10}N_4SO)].PF_6$ **(1)**	15.79(16.22)	2.97(3.23)	9.02(9.46)	5.14(5.41)
$[(CH_3CH_2)_3PAu(I)(C_5H_{10}N_4SO)].PF_6$ **(2)**	20.77(20.83)	3.85(3.97)	9.06(8.83)	5.13(5.06)
$[\{C(CH_3)_3\}_3PAu(I)(C_5H_{10}N_4SO)].PF_6$ **(3)**	28.81(28.42)	4.91(5.19)	7.96(7.80)	4.85(4.46)
$[(Ph_3)_3PAu(I)(C_5H_{10}N_4SO)].PF_6$ **(4)**	35.37(35.49)	3.02(3.24)	7.16(7.20)	3.96(4.12)

Table S2. Mid FT-IR wavenumbers (cm^{-1}) of ligand and gold(I) complexes (**1-4**)

**Compound**	ν(OH)	ν(NH_2)	ν(NH)	ν(C=N-OH)	ν(C=N)	ν(-N-C=S)
**Ligand(TBO)**	3415	3264,3238	3156	1598	1494	1374, 1286, 845
**1**	3427	3248, 3222	3165	1605	1507	1365, 1294, 845
**2**	3429	3247, 3220	3165	1607	1508	1367, 1297, 842
**3**	3429	3248, 3220	3165	1607	1508	1370, 1297, 838
**4**	3428	3246, 3221	3158	1605	1507	1367, 1295, 841

Table S3: ^{31}P NMR chemical shifts of phosphanegold(I) precursors and their complexes with TBO ligand in DMSO.

**Compound**	**^{31}P**	**δ_{shift}**	**PF_6^-**
$(CH_3)_3PAuCl$	-9.662		
$[(CH_3)_3PAu(I)(C_5H_{10}N_4SO)]PF_6$ **(1)**	0.089	9.751	-143.591
$([(CH_3CH_2)_3PAuCl$	32.077		
$[(CH_3CH_2)_3PAu(I)(C_5H_{10}N_4SO)]PF_6$ **(2)**	36.899	4.822	-143.591
$[C(CH_3)_3]PAuCl$	91.155		
$[\{C(CH_3)_3\}_3PAu(I)(C_5H_{10}N_4SO)].PF_6$ **(3)**)	97.021	5.866	
$Ph_3PAu(I)Cl$	33.818		
$[Ph_3PAu(I)(C_5H_{10}N_4SO)].PF_6$ **(4)**	36.792	2.974	-143.591

FT-IR spectra data

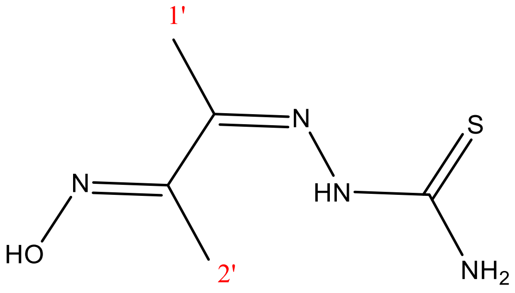

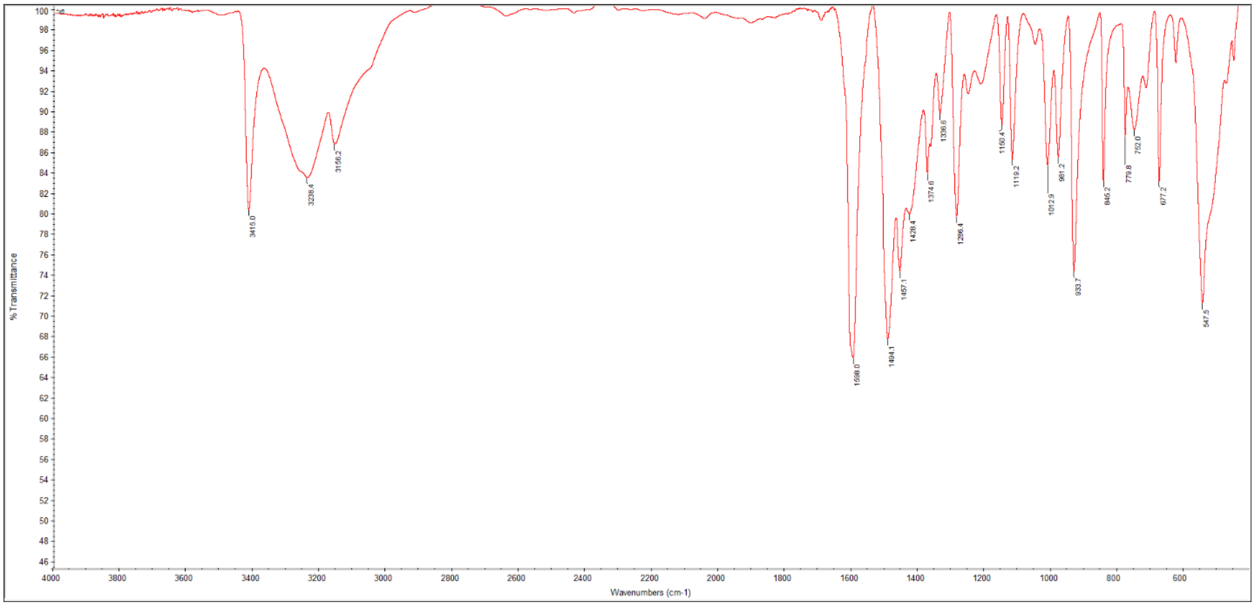

Figure S1: IR spectra of Ligand (**TBO**)

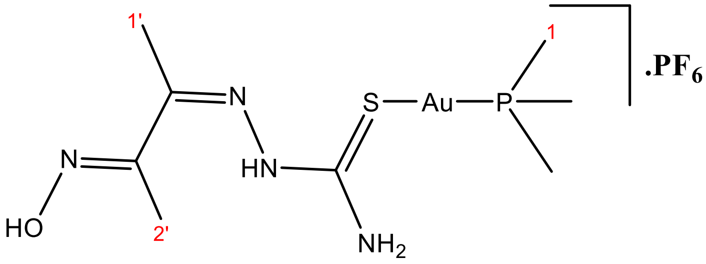

1

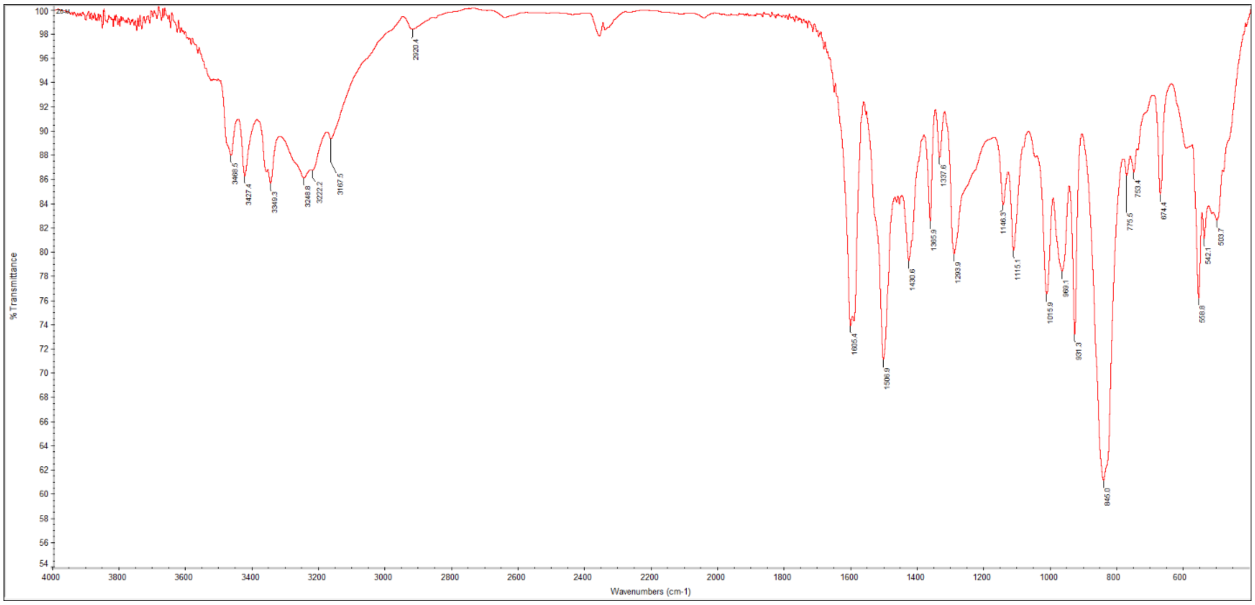

Figure S2: IR spectra of complex **1**

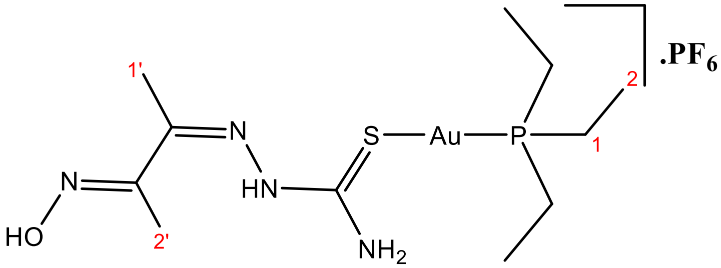

2

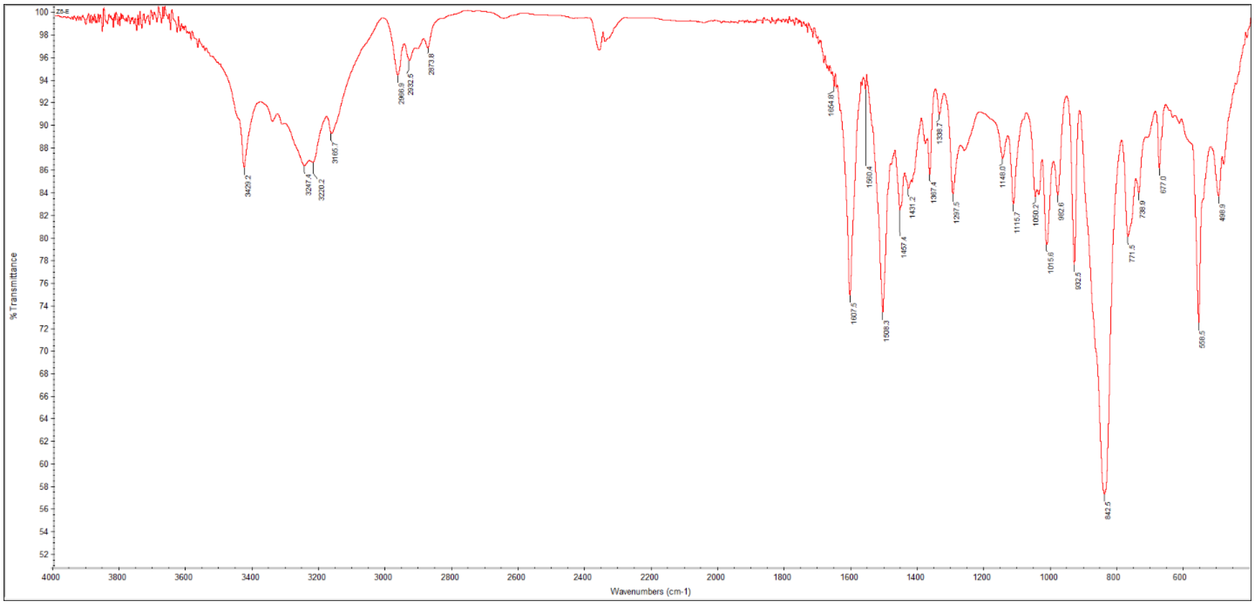

Figure S3: IR spectra of complex **2**

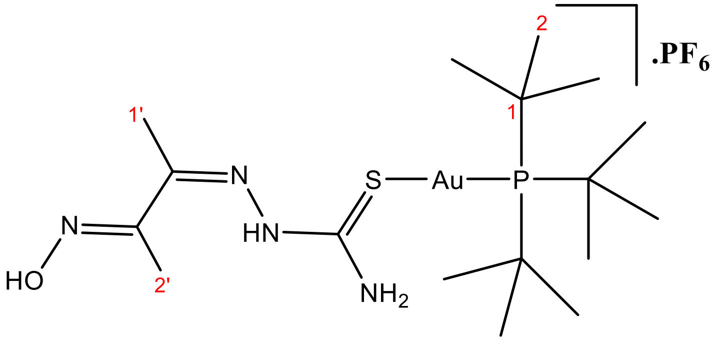

3

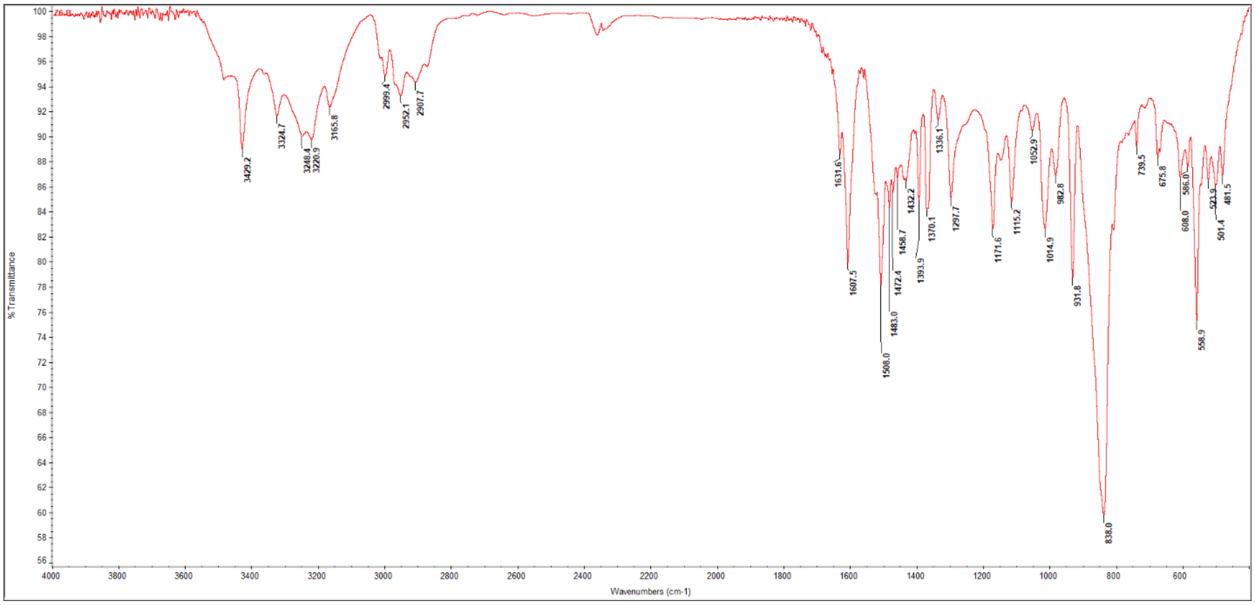

Figure S4: IR spectra of complex **3**

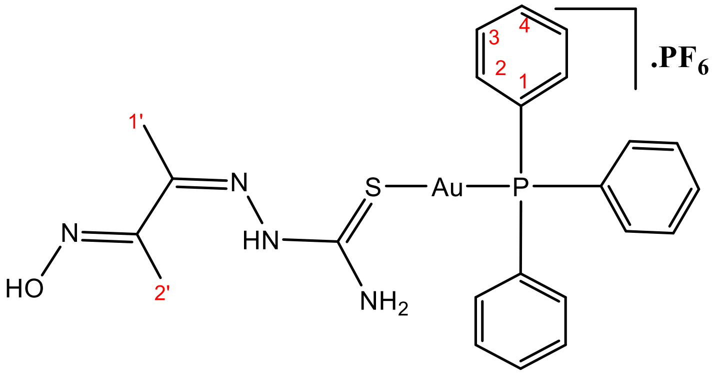

4

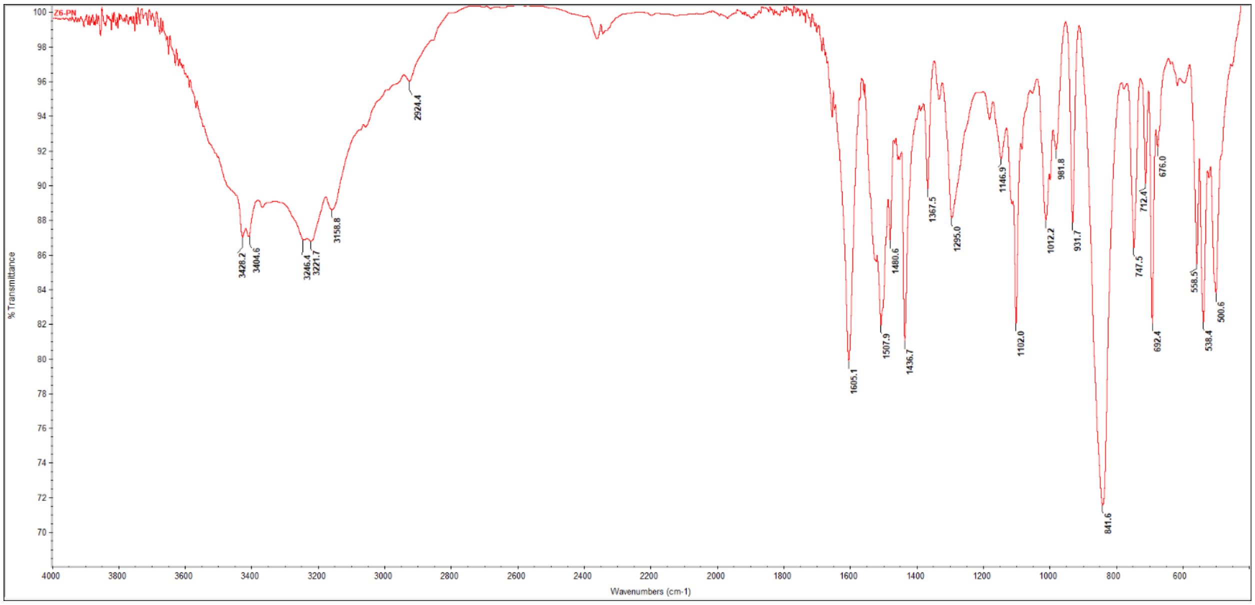

Figure S5: IR spectra of complex **4**

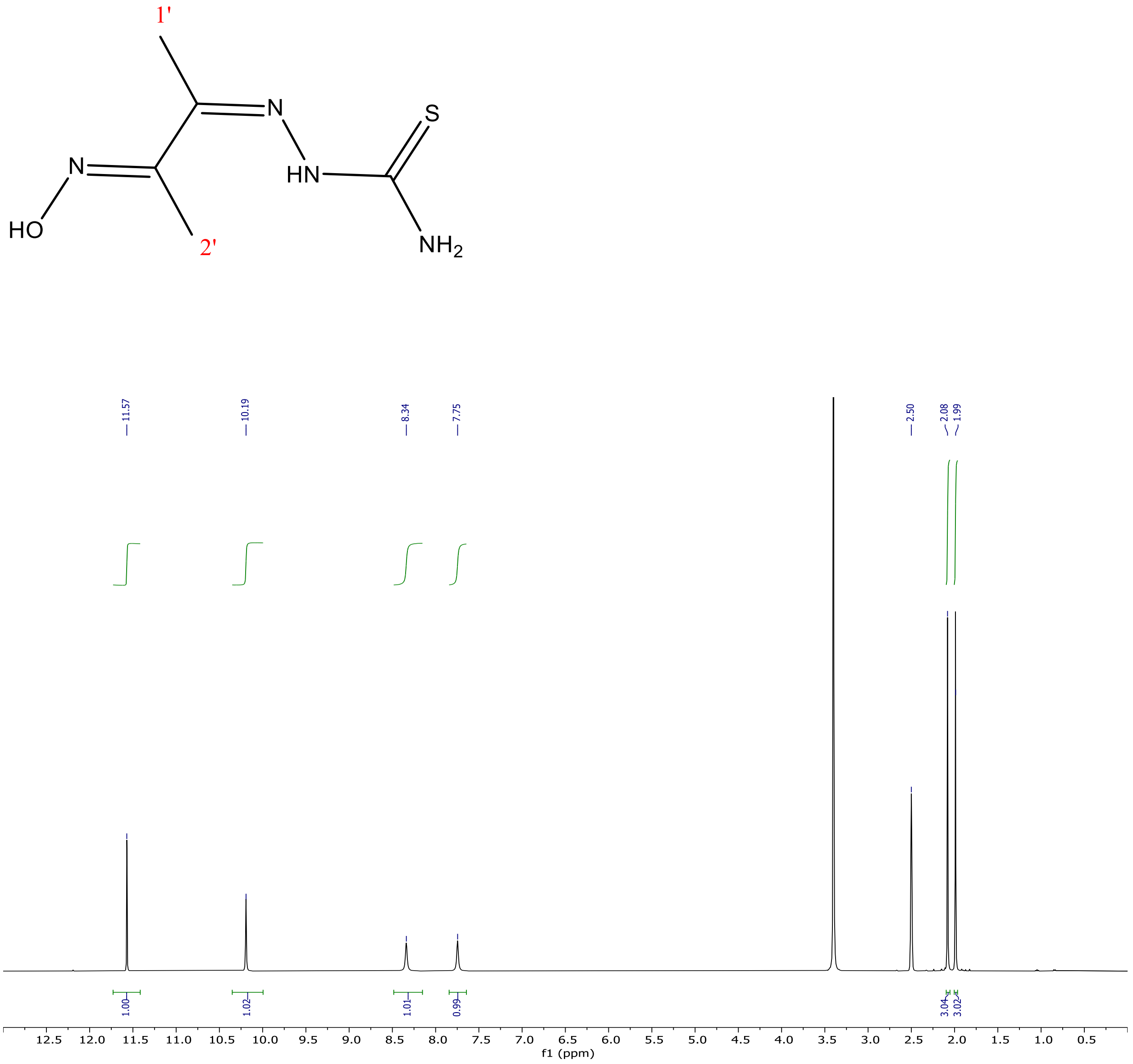

Figure S6: ^{1}H NMR spectrum of Ligand (**TBO**) in DMSO-d_6

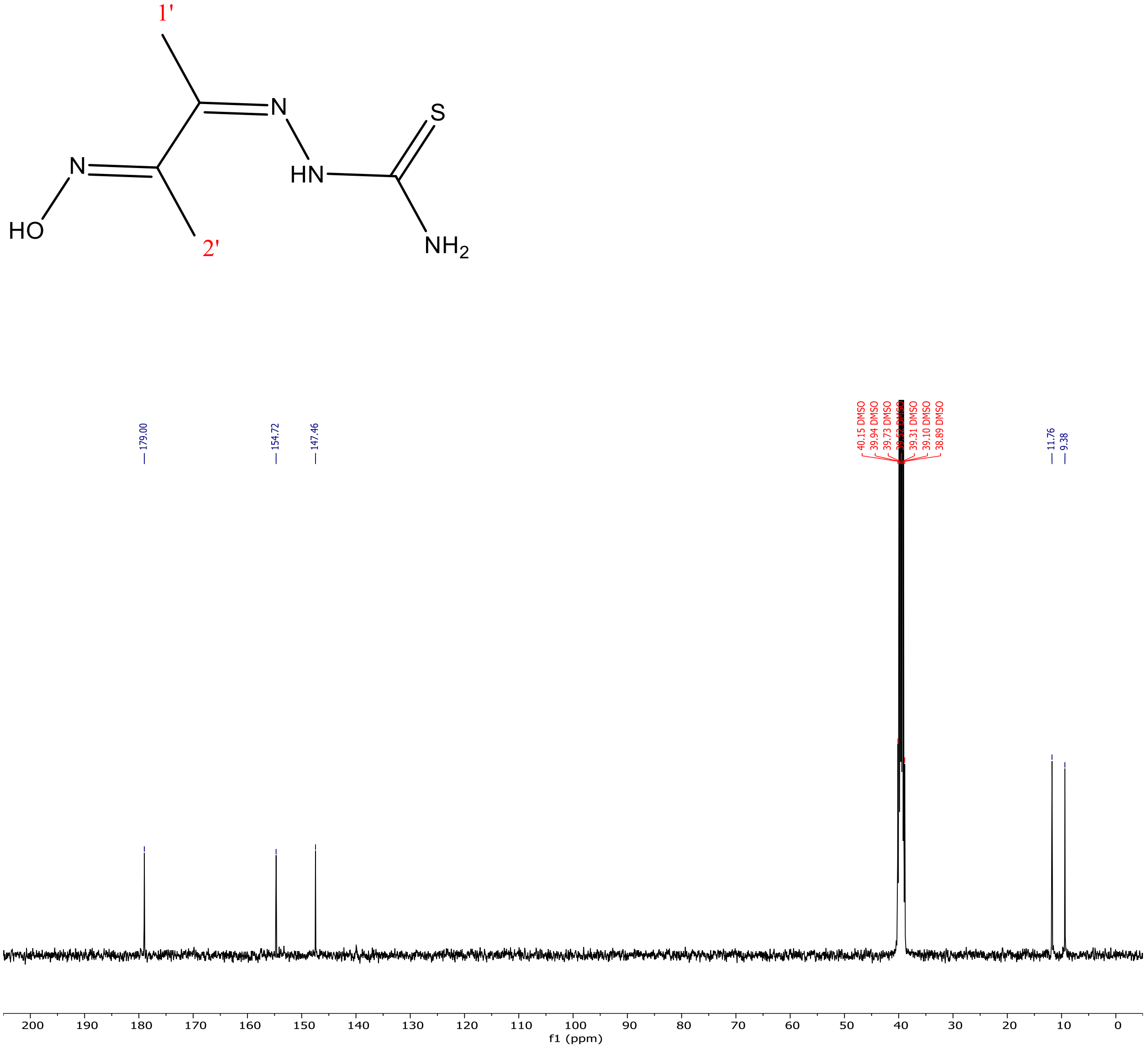

Figure S7: ^{13}C NMR spectrum of Ligand (**TBO**) in DMSO-d_6.

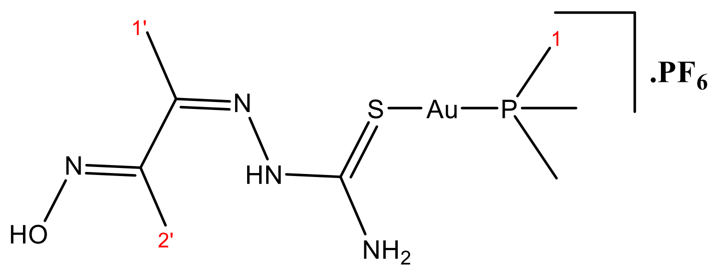

1

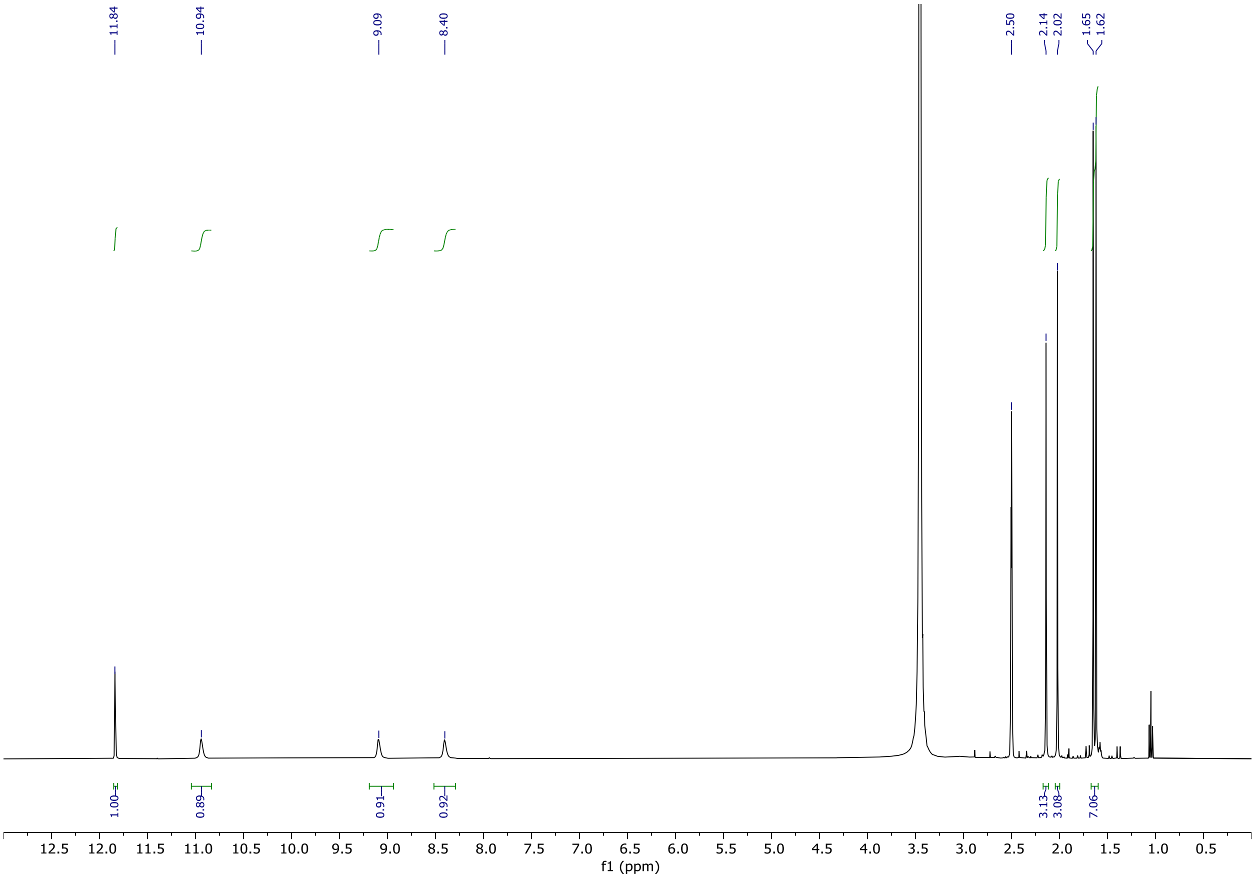

Figure S8: ^{1}H NMR spectrum of Complex **1** in DMSO-d6.

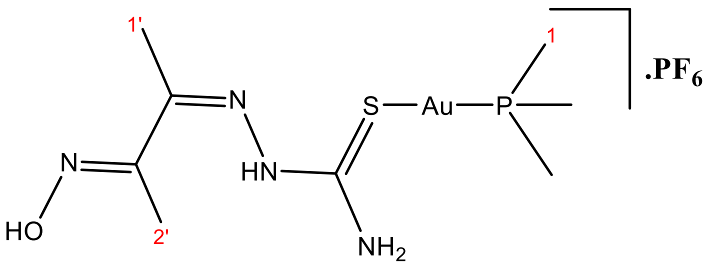

1

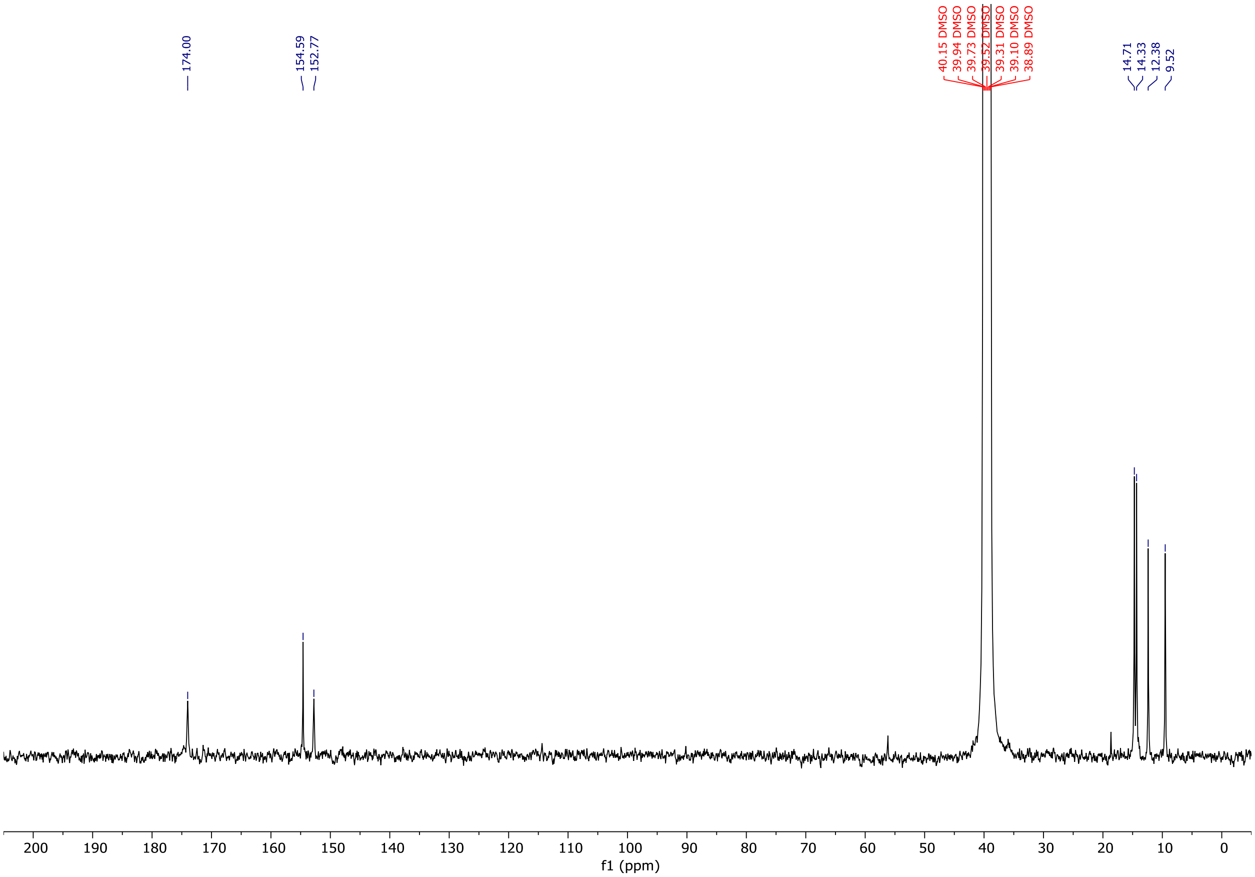

Figure S9: ^{13}C NMR spectrum of Complex **1** in DMSO-d6.

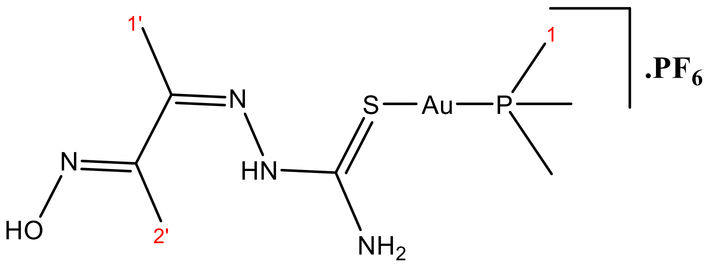

1

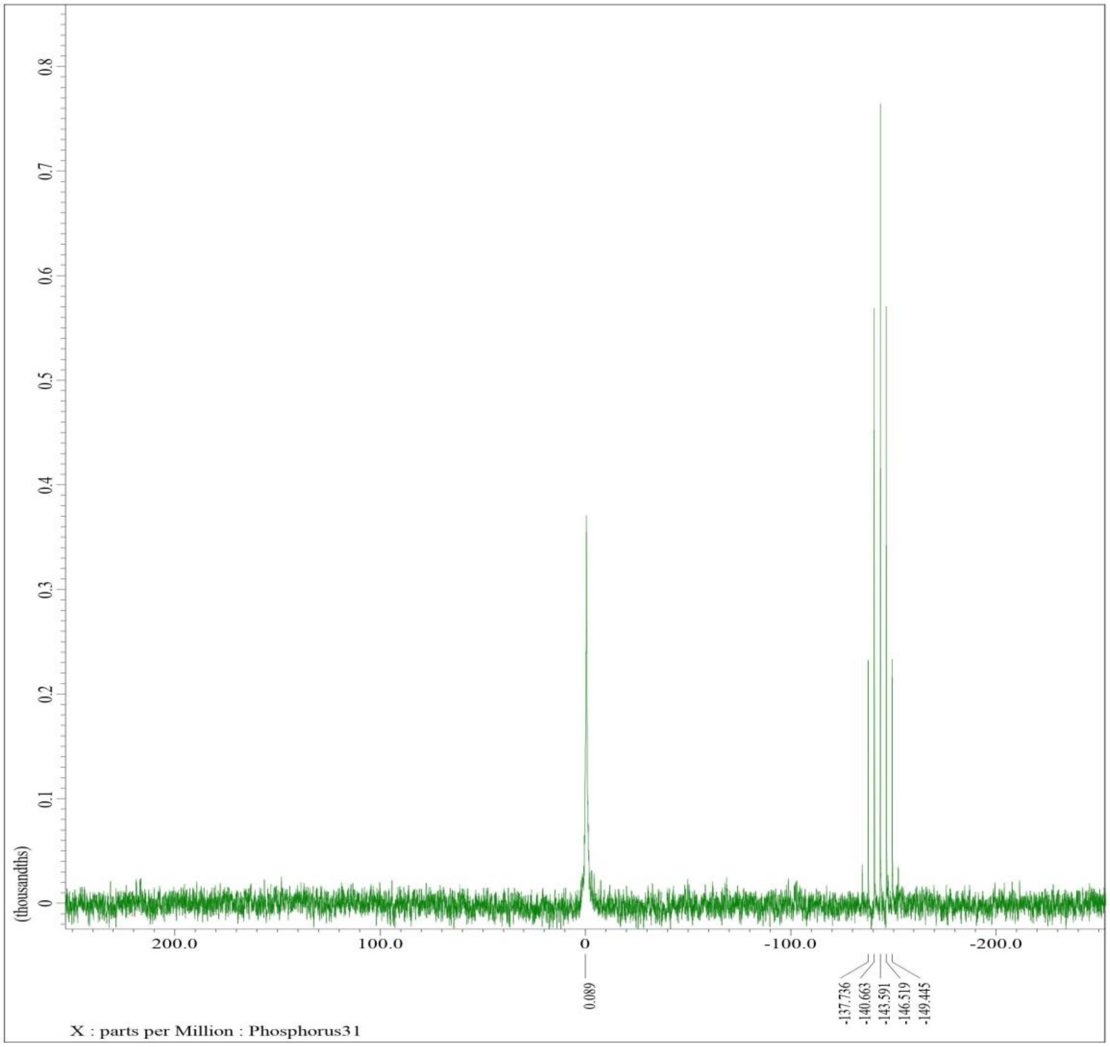

Figure S10: ^{31}P NMR spectrum of complex **1** in DMSO-d6.

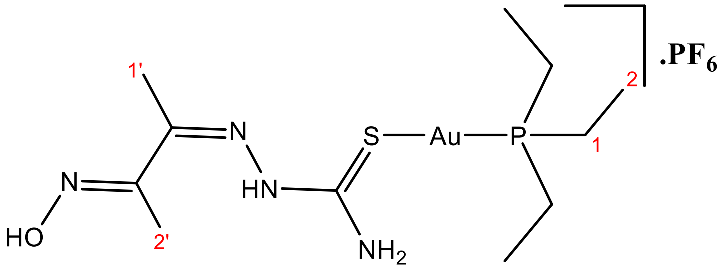

2

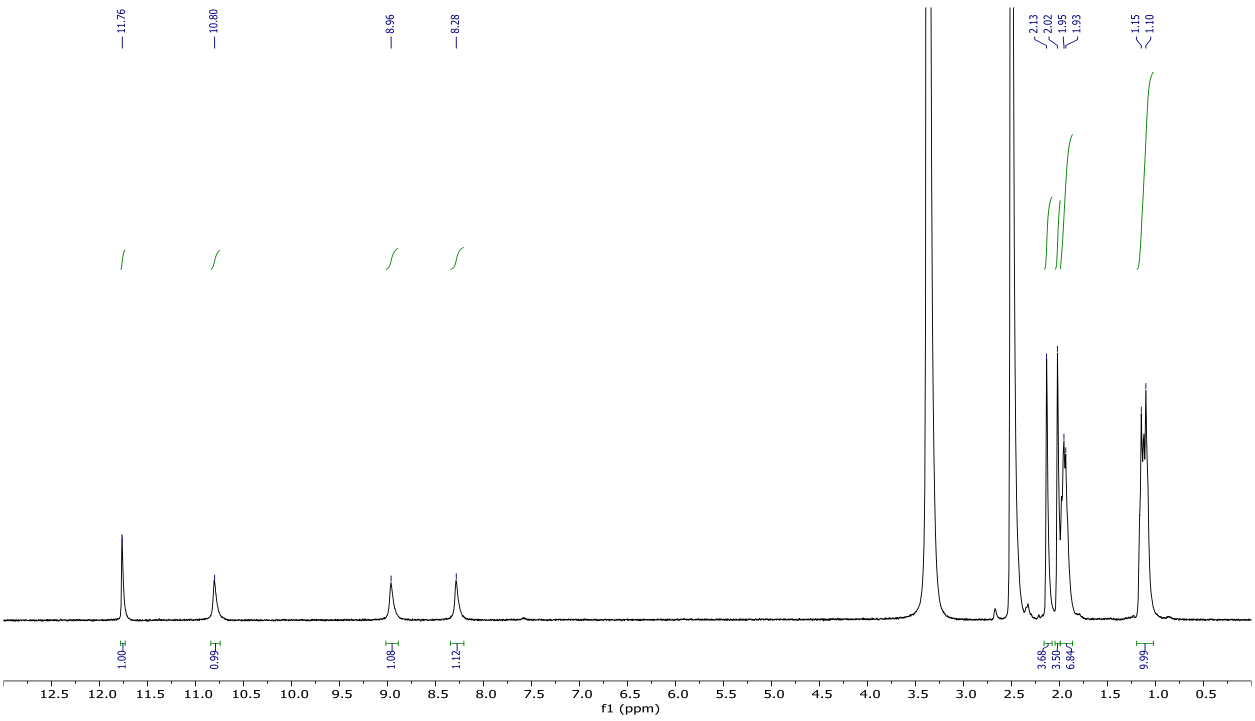

Figure S11: ^{1}H NMR spectrum of Complex **2** in DMSO-d6.

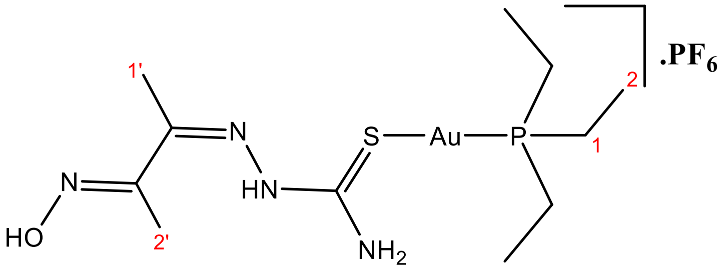

2

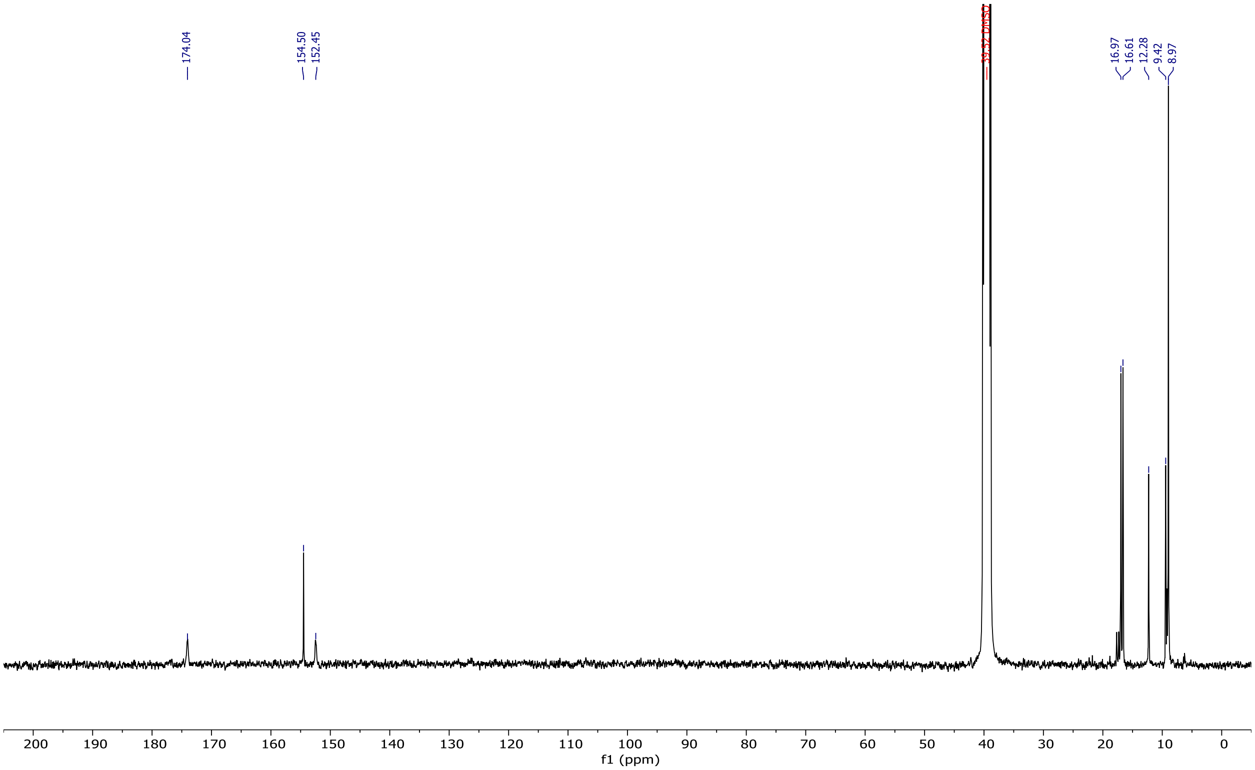

Figure S12: ^{13}C NMR spectrum of Complex **2** in DMSO-d6.

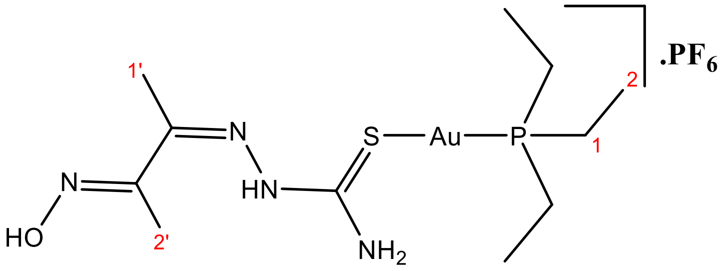

2

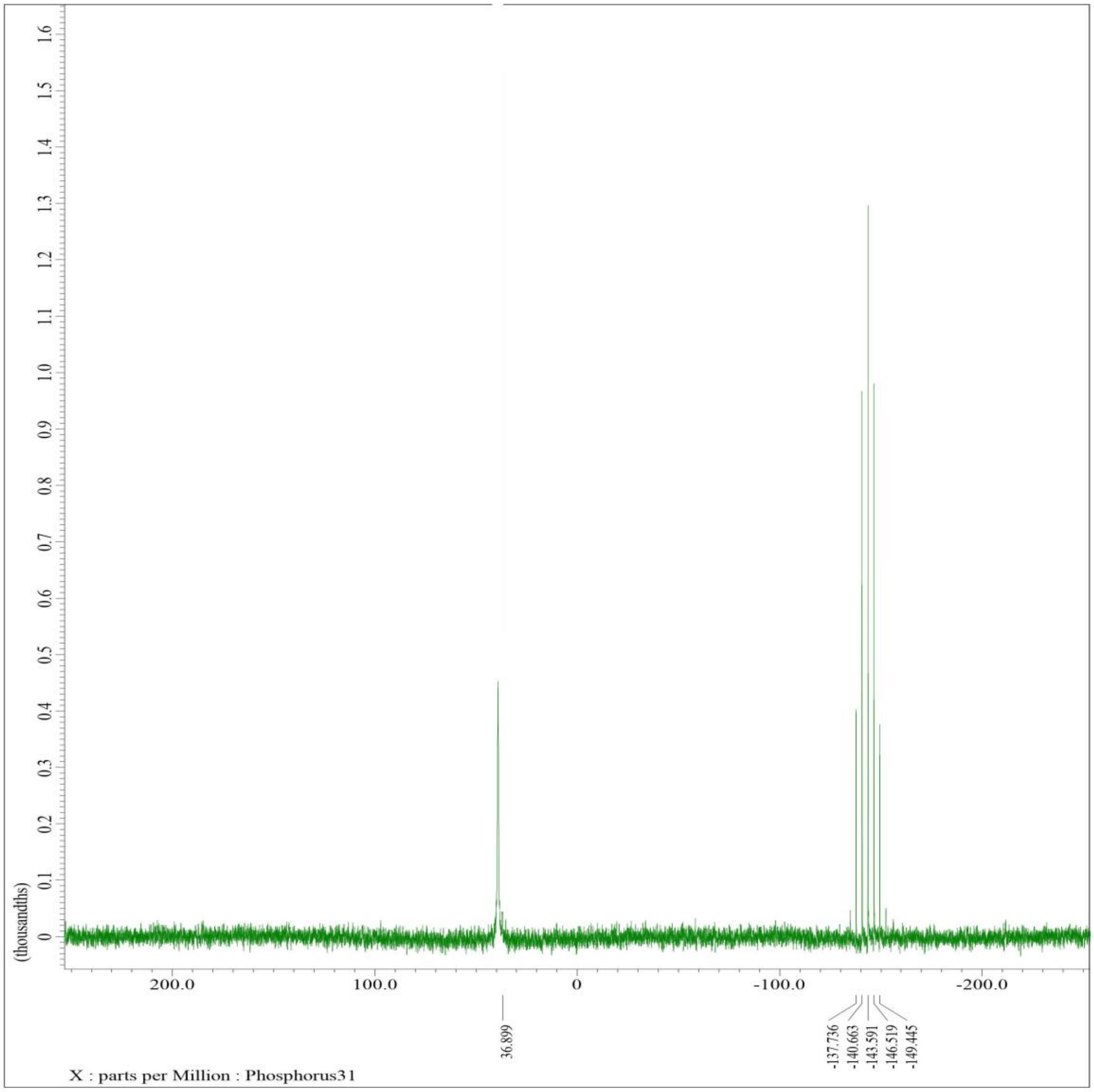

Figure S13: ^{31}P NMR spectrum of complex **2** in DMSO-d6.

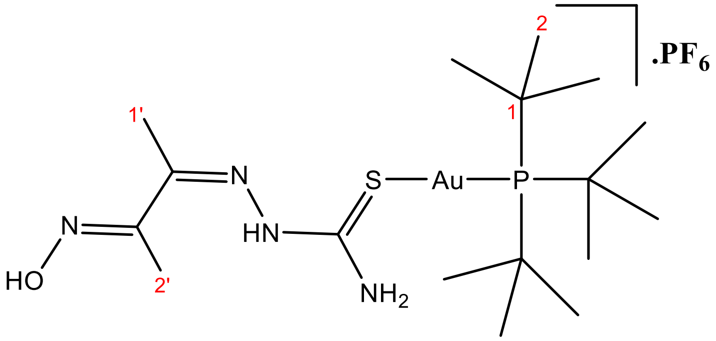

3

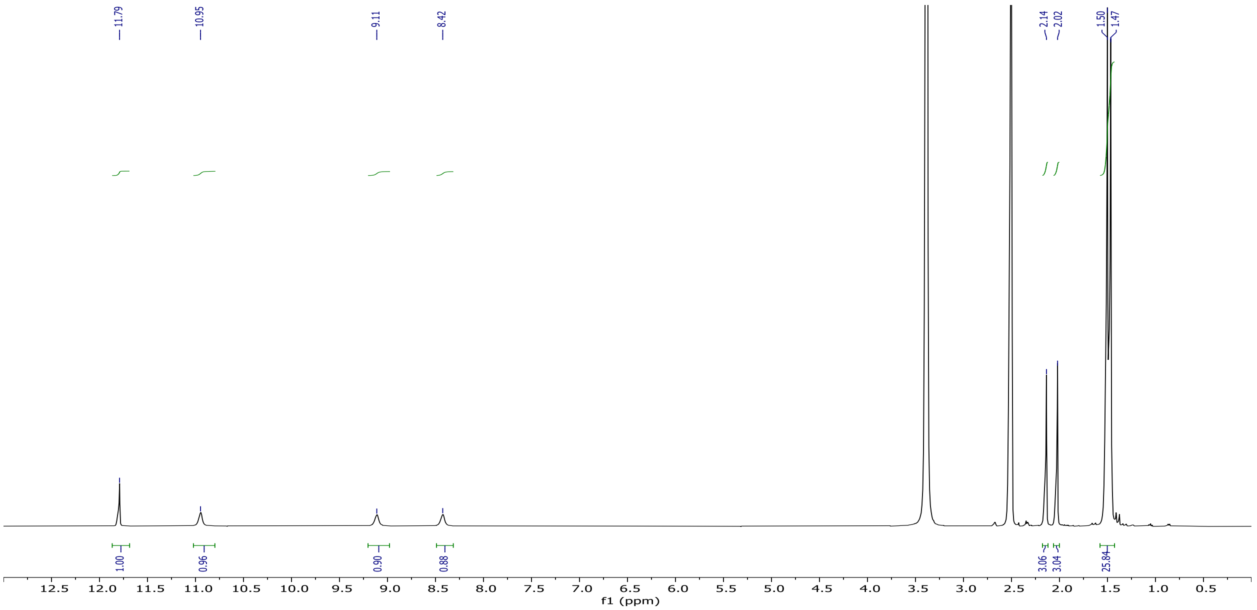

Figure S14: ^{1}H NMR spectrum of Complex **3** in DMSO-d6.

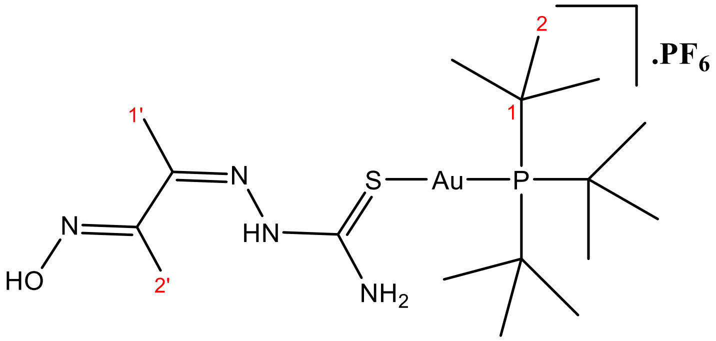

3

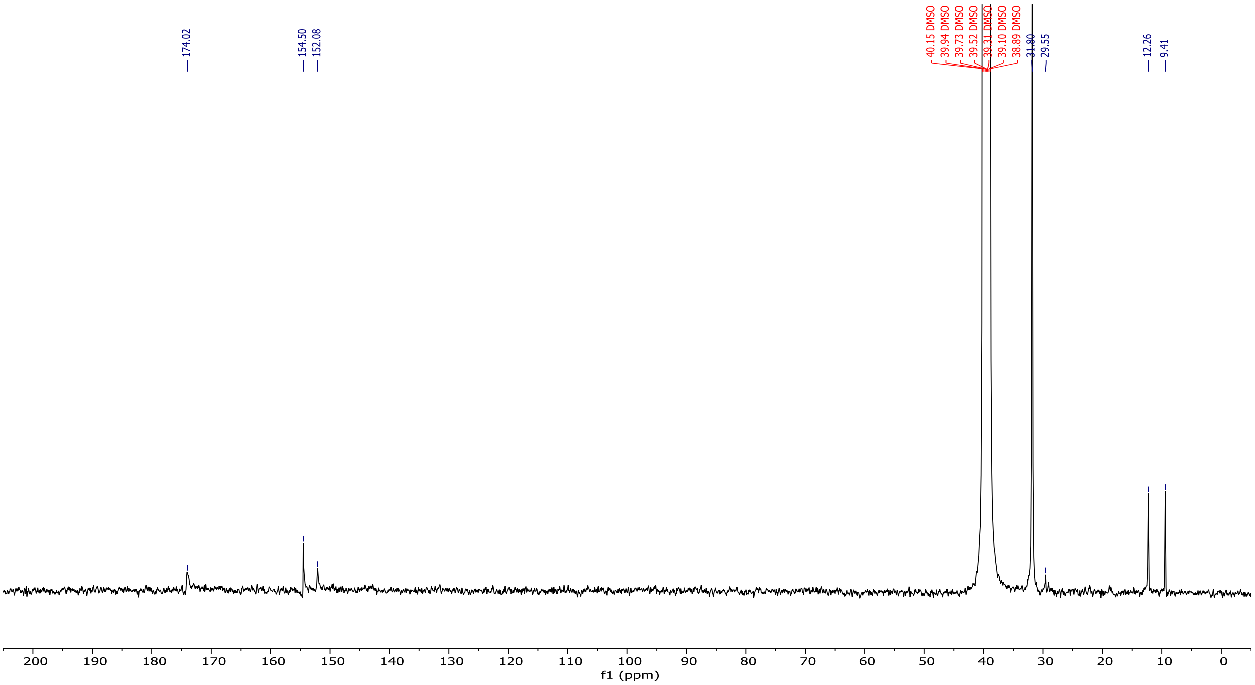

Figure S15: ^{13}C NMR spectrum of Complex **3** in DMSO-d6.

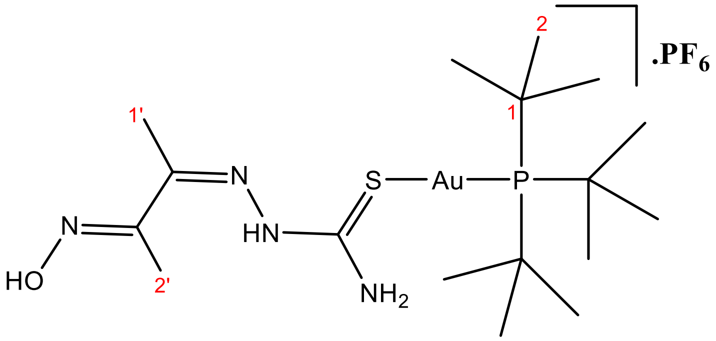

3

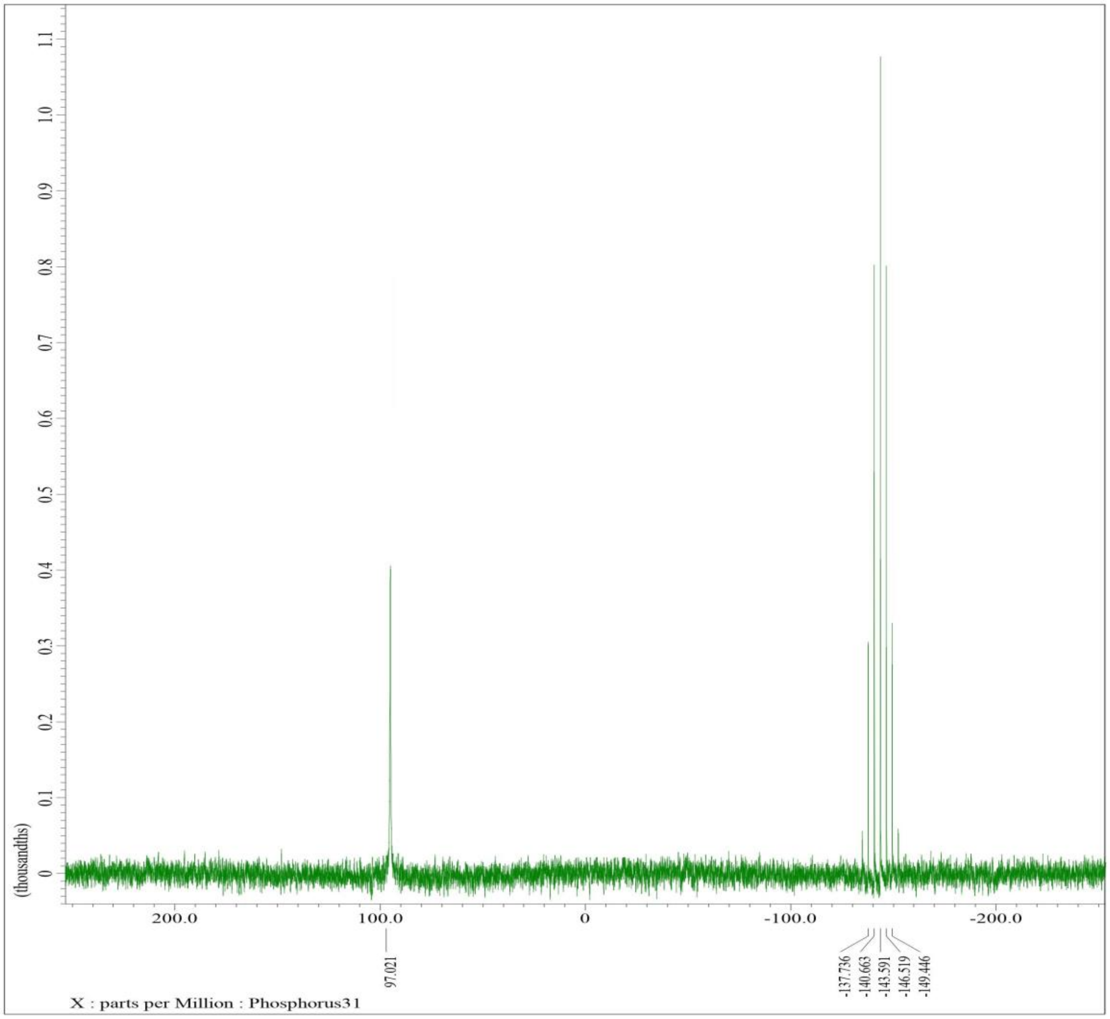

Figure S16: ^{31}P NMR spectrum of complex **3** in DMSO-d6

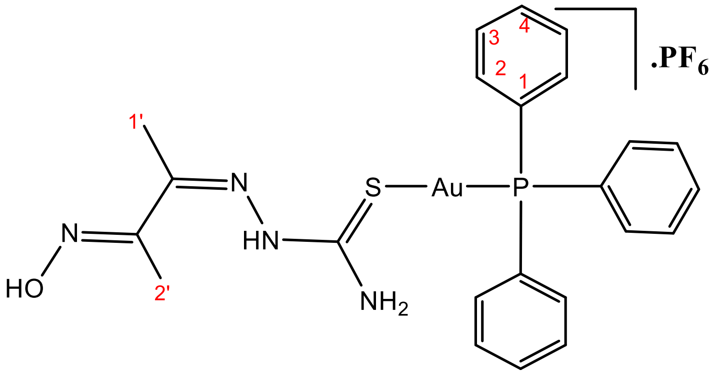

4

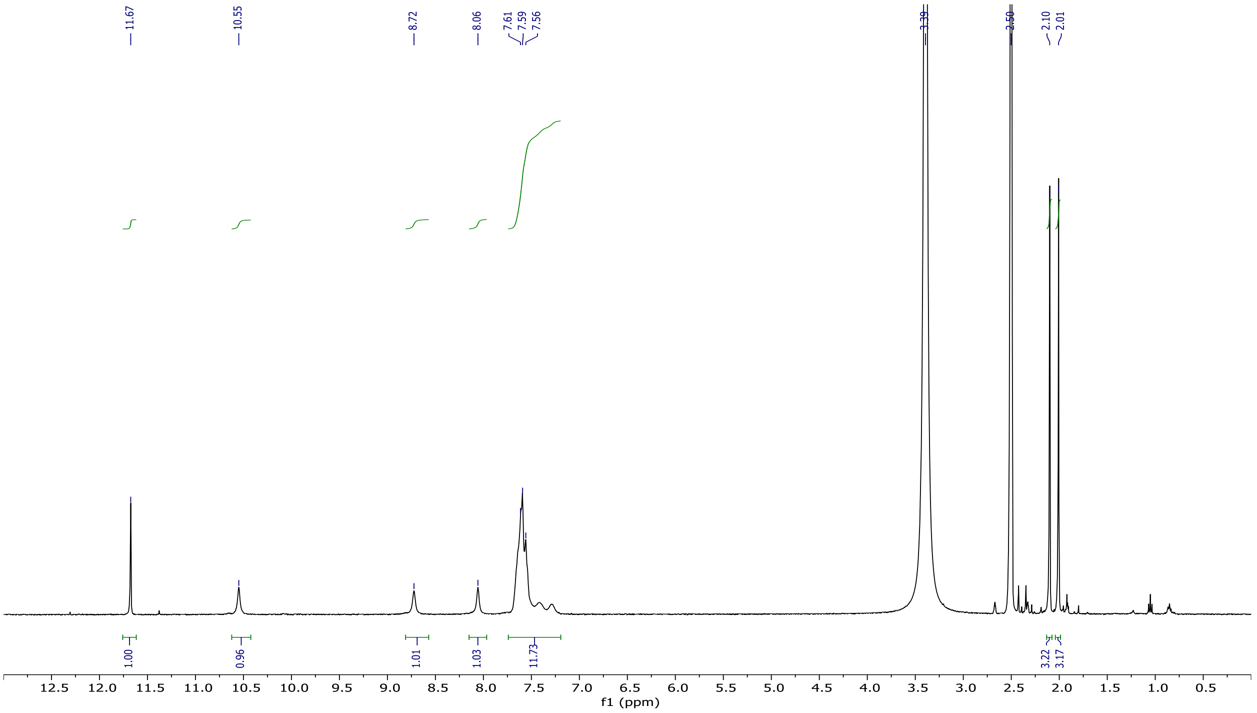

Figure S17: ^{1}H NMR spectrum of Complex **4** in DMSO-d6.

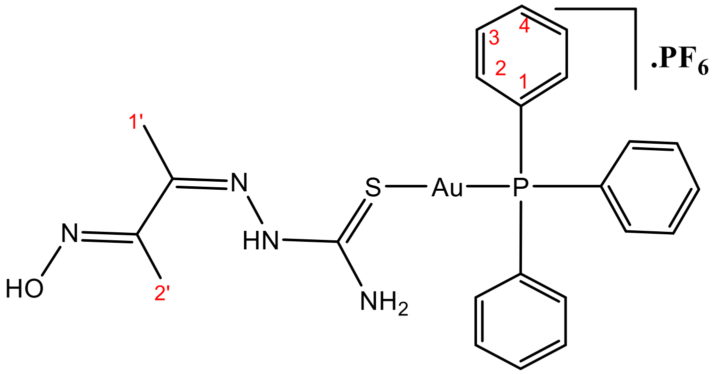

4

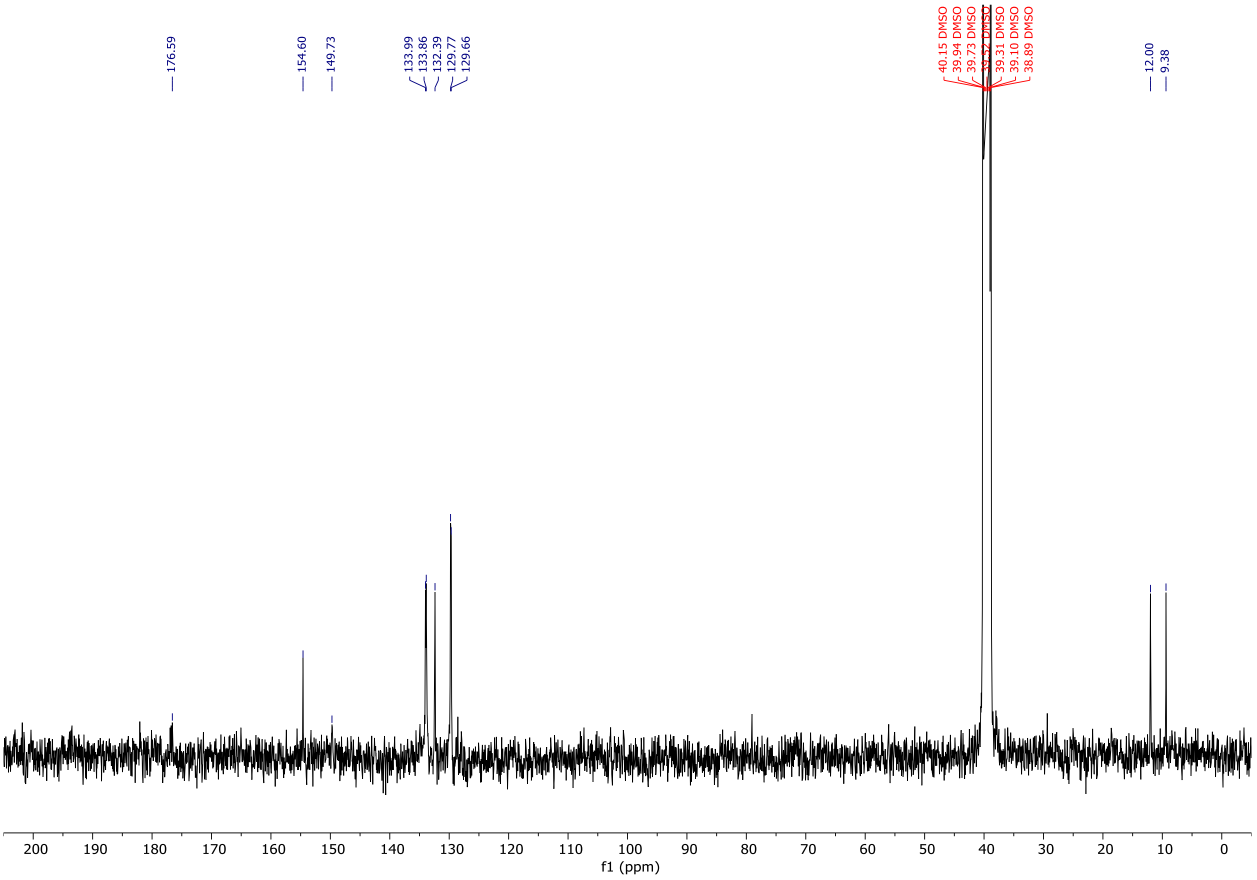

Figure S18: ^{13}C NMR spectrum of Complex **4** in DMSO-d6.

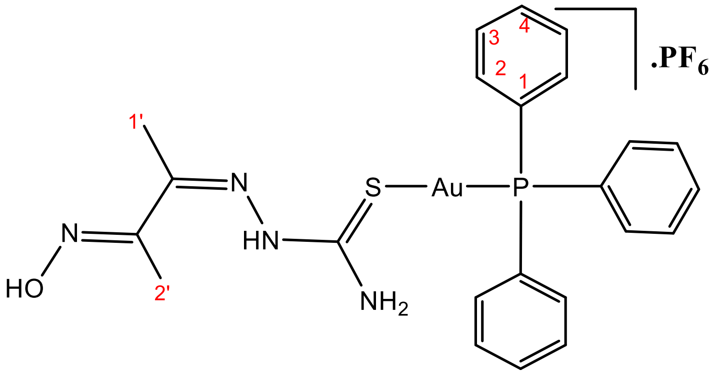

4

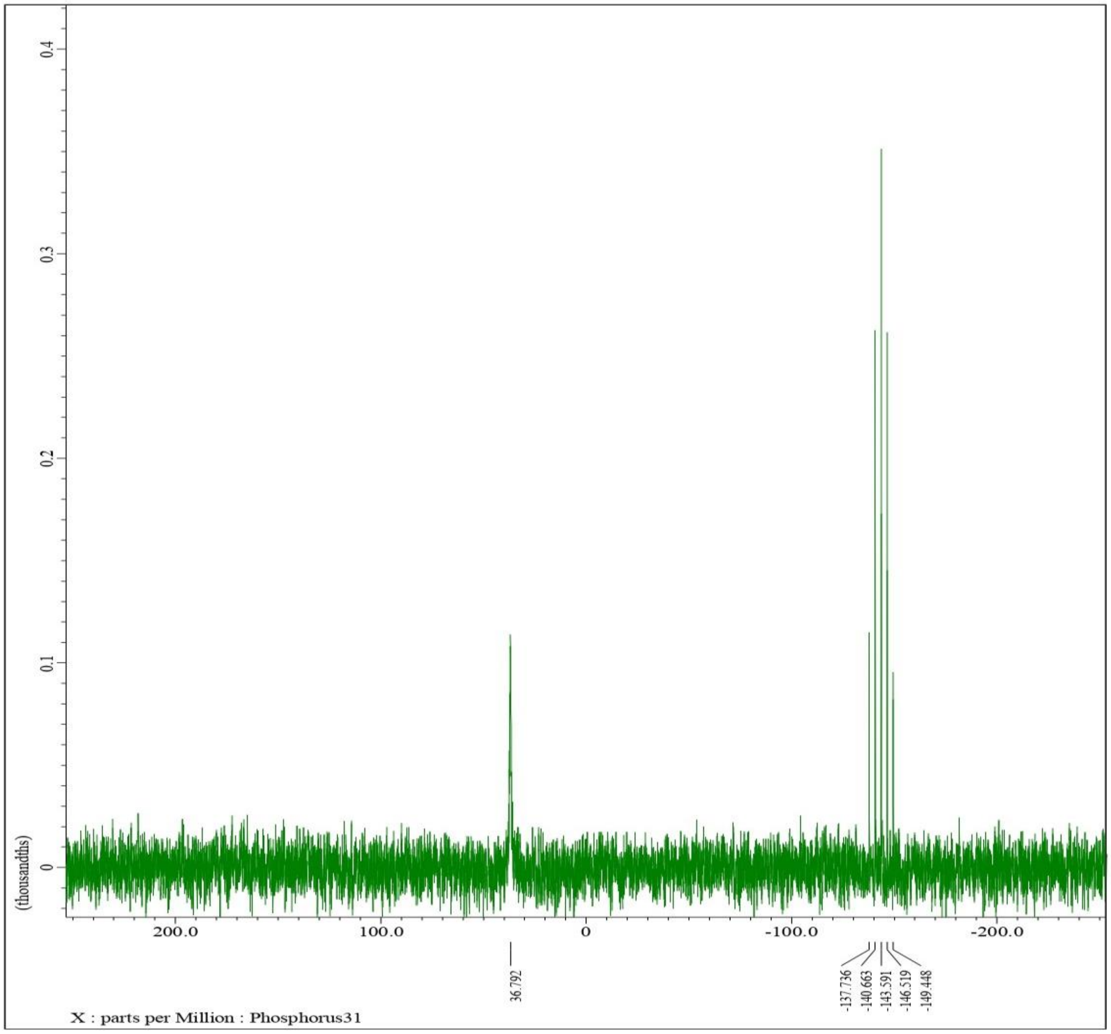

Figure S19: ^{31}P NMR spectrum of complex **4** in DMSO-d6

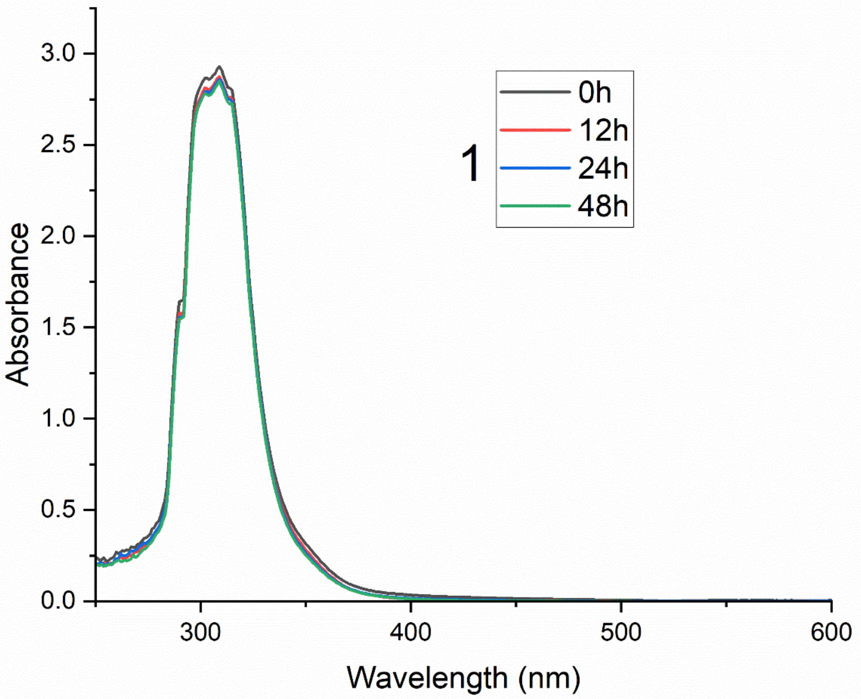
3.0
2.5
2.0
1.5
1.0
0.5
0.0
Absorbance
300
400
500
600
Wavelength (nm)
0h
12h
24h
48h
1

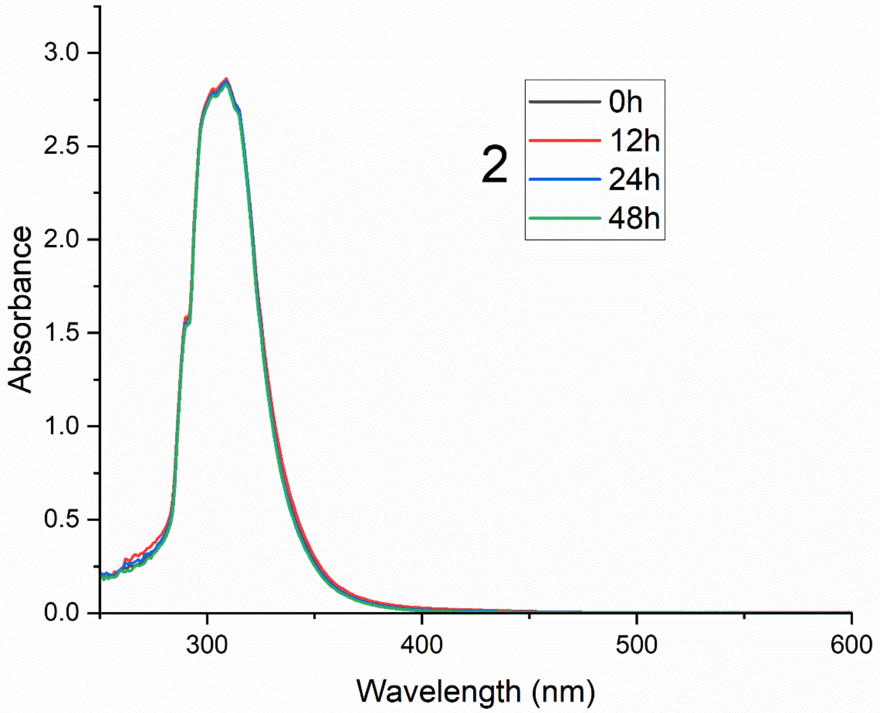
3.0
2.5
2.0
1.5
1.0
0.5
0.0
Absorbance
300
400
500
600
Wavelength (nm)
0h
12h
24h
48h
2

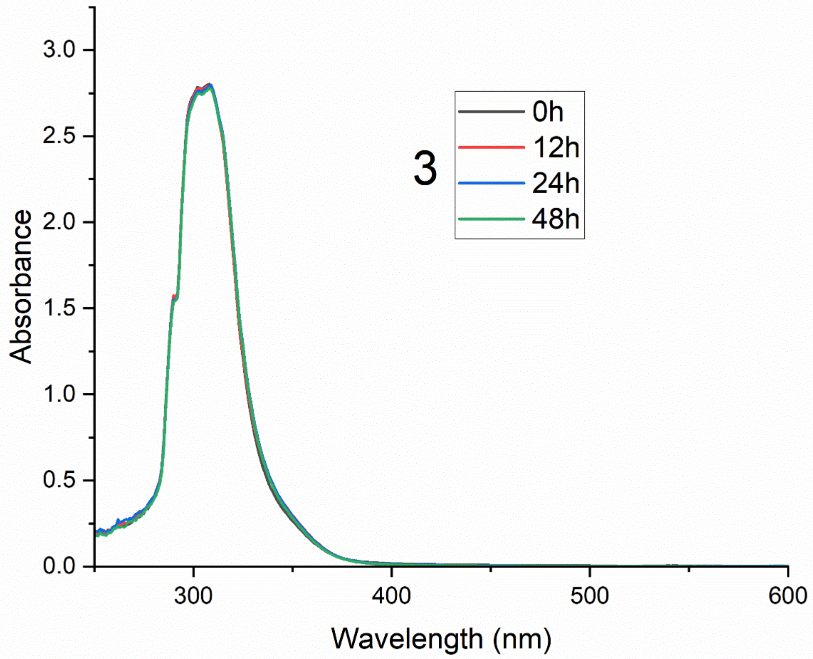

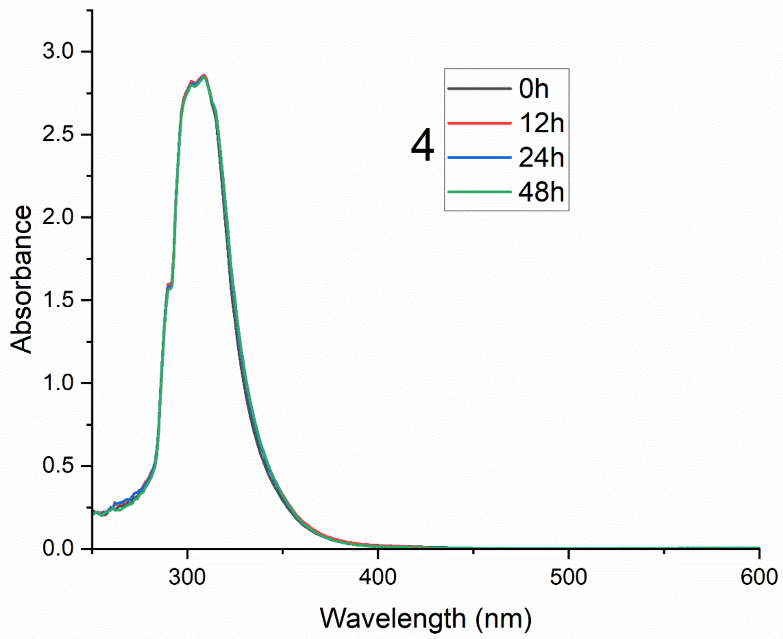

Figures S20: UV-Vis spectra of (0.25 mM, 10 mL) complexes stability (1-4) at the different timing in the solution of DMSO.

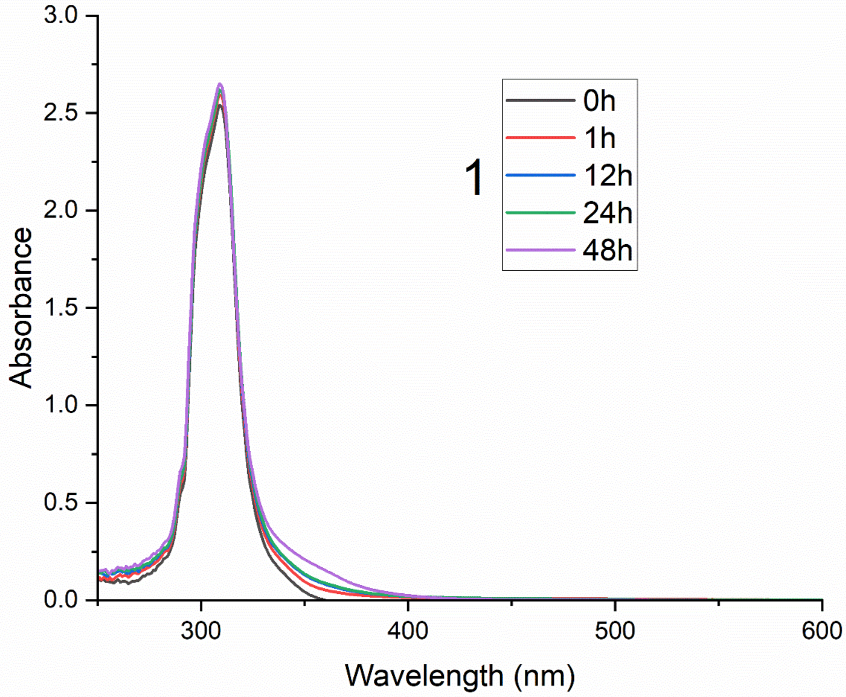
3.0
2.5
2.0
1.5
1.0
0.5
0.0
Absorbance
300
400
500
600
Wavelength (nm)
0h
1h
12h
24h
48h
1

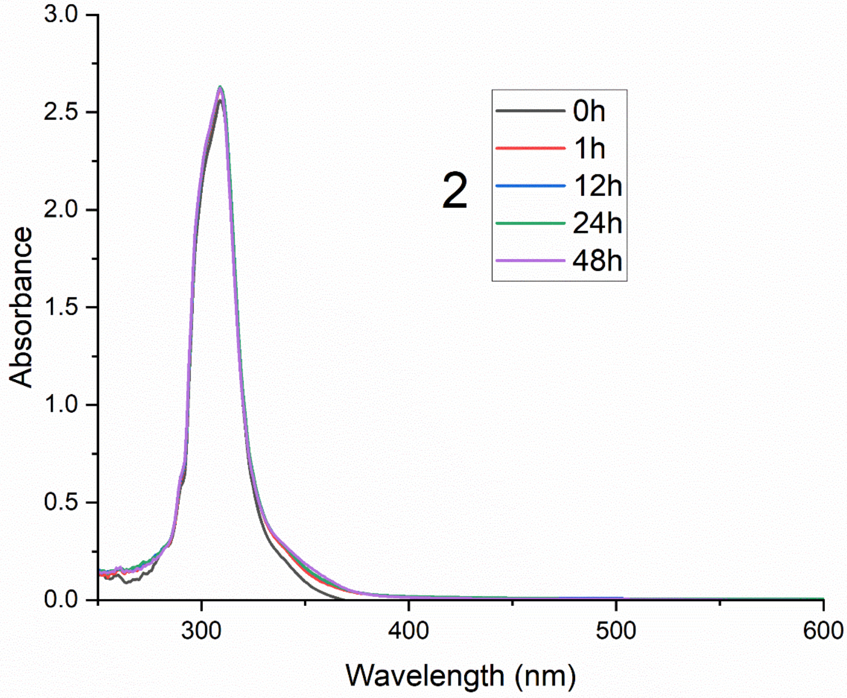
3.0
2.5
2.0
1.5
1.0
0.5
0.0
Absorbance
300
400
500
600
Wavelength (nm)
0h
1h
12h
24h
48h
2

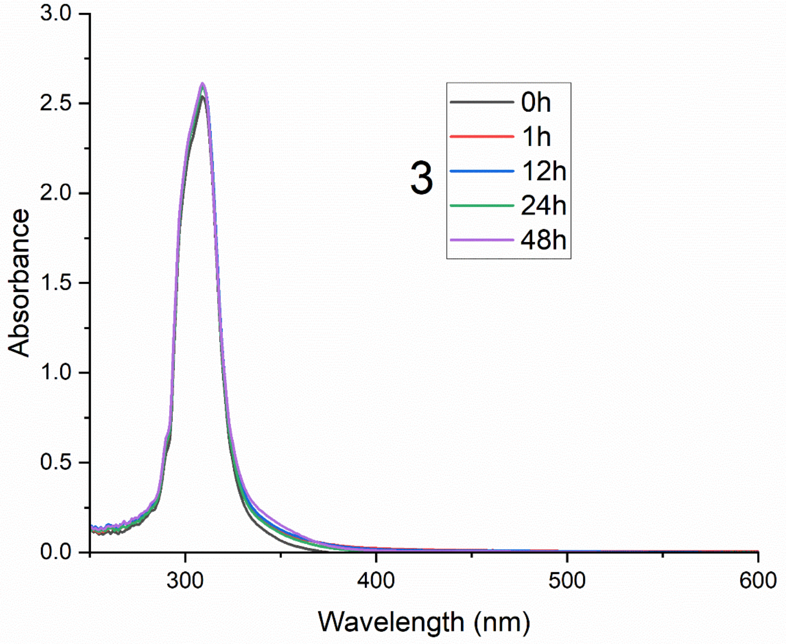

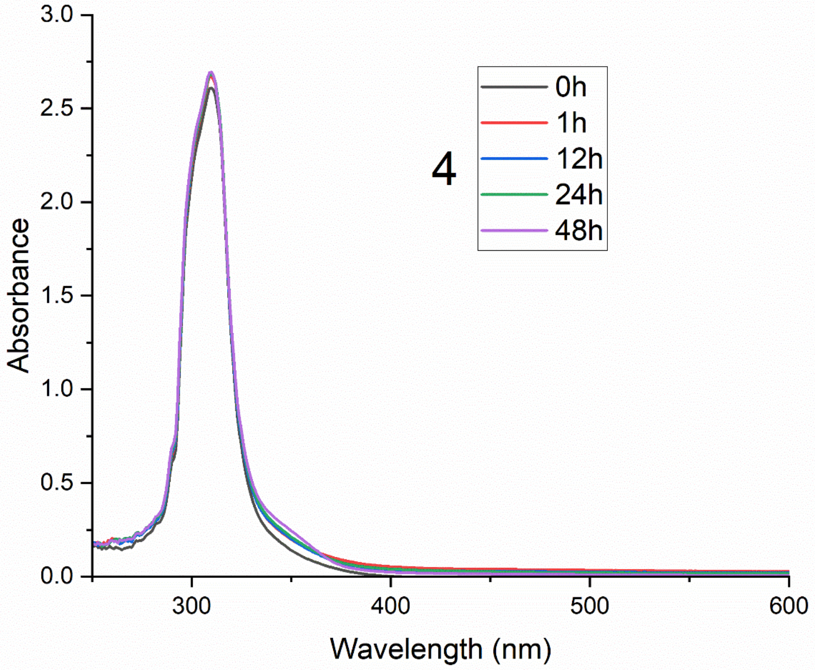

Figures S21: UV-Vis spectra of (0.25 mM, 10 mL) complexes stability (1-4) at the different timing in the solution of EtOH:H20 (1:1).
